# The Dilemma of Peri-Procedural Warfarin Management: A Narrative Review

**DOI:** 10.1177/10760296211012093

**Published:** 2021-11-29

**Authors:** Islam Eljilany, Ahmed El-Bardissy, Hazem Elewa

**Affiliations:** 1 College of Pharmacy, QU Health, Qatar University, Doha, Qatar; 2 Department of Pharmacy, Hamad General Hospital, Hamad Medical Corporation, Doha, Qatar

**Keywords:** vitamin K antagonist, bridging, peri-procedural management, survey, Qatar

## Abstract

Periprocedural vitamin K antagonist management is a complex process and inherently entails multiple clinical issues. Marked variations have been reported in different aspects of this process. These differences were noted at the clinician and institutional levels owing to the lack of evidence-based data leading to many discrepancies in decision-making. This review aims to address the gap of vitamin K antagonist periprocedural management acknowledged by previously published prescribers’ questionnaires. One of the components of this process is “bridging,” which aims to provide minimal interruption of the anticoagulation period through the use of heparin products. Recent studies showed that bridging is increasing bleeding risk. Secondly, interruption decision relies on the classification of thromboembolism risk which depends on trials that did not include patients with atrial fibrillation. Thirdly, the interruption duration is different among different International normalization ratio levels, which strengthens the difference in the clinical practice of preoperative vitamin K antagonist management. Lastly, the resumption of a vitamin-K antagonist after surgery has many scenarios according to the procedure and patient risk of bleeding. Vitamin-K antagonist periprocedural management is complicated due to individual practice and the lack of strictly implemented institutional standardized protocols to guide, manage and evaluate the process.

## Introduction

The perioperative management of patients receiving vitamin K antagonist (VKA) therapy is a common clinical dilemma. VKA regimens can be continued, interrupted, or replaced with other parenteral anticoagulants (ACs), namely “bridging.”^1^ The issue is always complicated with the small overlapping line between thrombotic and bleeding risks. An initial consideration is to avoid or delay the procedure until the VKA administration is no longer required, if at all possible. If not, both operation and patient status benefits and risks should be thoroughly considered.^1^

Clinicians should assess the periprocedural risks of bleeding or thrombosis. For example, patients with venous thromboembolism (VTE) are deemed at high risk of thrombosis when they had VTE episodes within the last 3 months or had severe thrombophilia. This risk is considered moderate when VTE episodes are within 3-12 months or in cases with moderate to mild thrombophilia or active cancer. The risk of thrombosis is deemed low in patients with VTE within >1 year without any other apparent risk factors.^1^ On the other hand, thrombotic risk assessment for patients with non-valvular atrial fibrillation (AF) and are at risk of stroke should be based on a CHA_2_DS_2_-VASc score. High scores (≥7) indicate a high thrombotic risk, while scores of 5-6 and 1-4 indicate moderate or low thrombotic risks, respectively.^2^ Subsequently, clinicians are required to assess whether there is an evident necessity to interrupt VKA to avoid the potential bleeding risks. If VKA interruption is deemed necessary, then a bridging decision should be made to consider thrombotic/bleeding risks, patient inconvenience and cost.

The objective of this review is to address the gap of VKA periprocedural management, which is acknowledged by previously published prescribers’ surveys through a comprehensive assessment of the published surveys on MEDLINE with PubMed interface.

## Review of Surveys Performed on the Periprocedural Management Based on Surgery Type

### Patients Undergoing Urological Surgeries

In 1999, a study was conducted in the United Kingdom. A postal survey was sent to urologists and radiologists concerning the practices and attitudes toward VKA and aspirin use in patients undergoing prostatic biopsies.^3^ Among 75% and 65% of responded radiologists and urologists preferred stopping VKA 3 days before biopsies by most participants, with a range between 1 and 8 days. Notably, 52% of the urologists did not indicate the existence of a specific protocol regarding preoperative management of ACs and the same percentage of participants were unaware about cases being postponed owing to patients unexpectedly receiving VKA. The study’s main finding is that the safe International normalization ratio (INR) level was widespread to proceed with biopsy (1.2-2.0). Another online questionnaire was emailed to urologists regarding their practice of VKA use before and after urological procedures, including minor surgeries, such as circumcision and biopsy, endoscopic procedures, and major surgeries, such as open radical prostatectomy and radical cystectomy.^4^ Approximately half of them responded, showing wide variations in their responses. For example, the range of preoperative discontinuation of VKA was 2-10 days, while no significant differences were noted according to the type of surgery. Heparin bridging was employed by 60% of the urologists. During the postoperative period, urologists restarted VKA 1-28 days after the procedures with a significant delay of reinitiating after major surgeries (4.38 ± 3.53 days) as compared to endoscopic (3.07 ± 3.52) and minor procedures (2.41 ± 2.31, *P* < 0.001). The authors concluded that procedure grad did not stimulus the VKA discontinuation preoperatively, but it influenced its re-initiation. Both studies presented that there is a wide discrepancy in preoperative management strategies for VKA with many urologists.

### Patients Undergoing Cardiovascular Surgeries

The anticoagulation regimens provided to patients undergoing cardiac rhythm device surgery were assessed among device implant physicians via postal questionnaires in Canada.^5^ The surveys presented 4 clinical scenarios to reveal the perceived risks of thromboembolic events based on a mechanical heart valve (MHV), a history of stroke, AF, and the risk factors (items) of the CHADS2 score. Furthermore, 6 managemental approaches were offered, including VKA interruption without bridging, 3 different bridging protocols, and ongoing VKA administration without interruption. Results showed that 83% of participants chose the ongoing VKA approach for patients with a low risk of thromboembolism. On the other hand, there were substantial variations regarding high-risk patients; heparin bridging was selected by 38%-72% of respondents while the remaining preferred VKA continuation. In this study, it is plausible that surgeons tended to pursue the risk of bleeding rather than the risk of thrombosis in the perioperative period since bleeding is rapidly detectable and manageable in such a patient population.

### Patients Undergoing Dental Procedures

A sample of general dental practitioners (GDPs) in Wales answered a questionnaire about their VKA peri-procedural management practices.^6^ Only 1% indicated that the patient should stop VKA before surgeries without consulting other medical practitioners. Notably, 34%, 30%, 10% and 10% of the responders considered normal INR upper limit at 2.5, 3.0, 3.5 and 4.0, respectively. This study showed that the practice of GDPs differed from the 2001 recommendation, and a number of them lacked the required knowledge.

In another cross-sectional study conducted among the Michigan Society of Oral and Maxillofacial Surgeons in the United States,^7^ 188 surveys were distributed to assess practice levels regarding patients receiving AC therapy. Dental procedures of different risks were indicated. VKA discontinuation was significantly predominant in high-risk procedures (70.5%) when compared to moderate (48.8%) and low-risk procedures (23.6%, *P* < 0.01). Indeed, these results were relatively surprising since VKA discontinuation in the low-risk group, which includes 1-5 simple extractions, was indicated even though INR values were maintained at the therapeutic levels (mean = 2.68). On the other hand, the practice of dental surgeons regarding moderate- (6-10 simple extractions, one quadrant alveolectomy, and 1 impacted extraction) and high-risk procedures (>10 simple extractions, >2 quadrant alveolectomy, and >2 impacted extraction) are not guided by evident literature investigations. Therefore, it seems that the current attitudes of dental surgeons are affected by the lack of uniformity that exists in the literature. The authors emphasized the need to conduct relevant prospective clinical trials that help create uniform guidelines.

### Patients Undergoing Cutaneous Surgeries

The significance of perioperative management of AC in patients undergoing cutaneous surgeries was initially presented in a case series of 2 patients who experienced a postoperative stroke after VKA discontinuation.^8^ Moreover, Kargi et al^9^ revealed that VKA-receiving patients are at significant risks of persistent bleeding, skin graft loss, and wound hematoma and infection during the procedures. However, minor surgeries could be performed while taking VKA with precaution. Therefore, in 2002, Kovich and Otley^10^ sent mailed surveys to assess the practice among 168 surgeons of the American College of Mohs Micrographic Surgery and Cutaneous Oncology. Most of the respondents (80%) employed VKA interruption, where they discontinued VKA 3 days preoperatively and continued it 1-2 days after the procedures. Besides, only 10% of the participants prescribed heparin bridging in the instance of VKA interruption. These findings were relatively inconsistent with the published recommendations at the time,^11^ which indicated VKA continuation during cutaneous surgeries without major risks and, if discontinued, heparin bridging should be considered in high-risk patients. Survey results also showed that only 15% of surgeons measured INR or prothrombin time preoperatively. This observation may be related to surgeons’ reliance on the latest results of the regularly performed laboratory investigations rather than requesting new ones.^10^

However, 3 years later, another survey of surgeons of the same organization showed different results. Kirkorian et al^12^ received completed surveys from 271 physicians (response rate 38%) regarding their practices of patients receiving AC and undergoing Mohs surgery, biopsy, excision, liposuction, and blepharoplasty. Although 62% of the participants indicated that ACs usually lead to significant bleeding during surgeries, 56% and 63% of them never discontinued VKA and aspirin during the procedures, respectively. In VKA interruption, less than half of physicians (42%) discontinue the medication 3 days preoperatively. Indeed, these results show a significant change in practice levels within a short period compared to the previous survey, with a remarkable shift toward VKA continuation. Notwithstanding the agreement between physicians’ attitudes and the recommended guidelines,^11^ the survey results underscore the urgent need to set the safety standard through solid recommendations.

More recently, Khadim et al^13^ investigated the practice of members of the British Association of Plastic Surgeons regarding perioperative AC management of patients undergoing cutaneous surgeries of the head and neck. Among 113 respondents, 43% discontinued VKA preoperatively, 33% decided based on INR values, while only 18% preferred to continue VKA. For the INR-dependent group, there was a controversy in decision-making, where VKA discontinuation was based on INR values over 3, 2.5, and 2 in 36%, 22%, and 36% of participants, respectively. Among VKA-interrupting respondents, the reasons for interruption were reducing intraoperative bleeding (73%), concerns about hematoma incidence and subsequent skin graft failure (66%). 34% of physicians had already experienced 1 or more severe complications owing to VKA interruption. Importantly, 34% of the participants indicated that their practices were based on local departmental policies, while only 6 respondents preferred to consult clinicians from related specialties regarding changing AC therapies. Overall, these results show significant variability in perioperative practices among plastic surgeons. This was associated with an unexpectedly high incidence of complications and, therefore, standard protocols are needed to address these issues.

### Patients Undergoing Ophthalmological Surgeries

Several studies assessed anticoagulation management of ophthalmological procedures. An early investigation was conducted in 2000 concerning all ophthalmic consultants and oculoplastic specialists in patients undergoing dacryocystorhinostomy, entropion, ectropion, or ptosis procedures in a local region in the United Kingdom (n = 62).^14^ At least half of the participants tended to interrupt VKA, with the most diminutive proportions reported cessation before entropion procedures and the highest proportion before dacryocystorhinostomy. Among those physicians, VKA was stopped at an average of 3 days (range 1-10 days) preoperatively. The majority of participants (93%) did not consult other relevant specialists about changing AC therapy, although a considerable proportion of them (54%) reported severe complications with either stoppage or continuation of VKA. Such complications included systemic metabolic episodes, hemorrhagic surgical complications, and mortalities (due to either brachial artery embolism, or cerebrovascular accidents).

Another questionnaire-based study was established among the Canadian Society of Cataract and Refractive Surgery to investigate their attitudes toward using VKA perioperatively.^15^ A total of 82 physicians responded (response rate: 74.5%) from different Canada regions. Approximately one-quarter (23.2%) of physicians discontinued VKA before surgeries with a range of 3-7 days. 82.6% resumed VKA on the first day, and the remainder continued it on the second day postoperatively. The small number of physicians interrupting VKA may be attributable to the remarkable advances accomplished in cataract surgery, rendering it a minimally invasive procedure. Of note, physicians who employed VKA interruption had more years of experience and performed surgeries less frequently when compared to their VKA-continuing counterparts. However, 9 patients experienced complications due to VKA interruption, including cerebrovascular accidents, deep vein thrombosis (DVT), transient ischemic attack, and death (in 1 patient). In the latter case, the patient stopped VKA 5 days before surgery. On the other hand, 15 patients experienced a hemorrhagic complication with VKA continuation, including peribulbar hemorrhage, retrobulbar hemorrhage, and hyphemia.

Continuing with cataract surgery, a more extensive study conducted in the United Kingdom showed similar outcomes with lower VKA discontinuation rates.^16^ Among a total of 535 cataract surgeons who participated in the survey, 69.3% were aware of the existence of VKA-specific departmental guidelines, and 98% indicated the importance of INR measurement perioperatively. Only 13.2% of participants stopped VKA preoperatively; most of them did so 2-3 days before surgeries (with a range of 1-14 days). It is worthy to note that VKA discontinuation was associated with a total of 18 complications, including cerebrovascular events, DVT, arterial embolism, Pulmonary embolism, myocardial infarction, and one reported mortality. Indeed, when compared to the previously mentioned study and other earlier studies,^15,17,18^ emphasized the gradual decreasing trend in VKA interruption before cataract surgeries. However, this agreed with the guidelines implied by the Royal College of Ophthalmologists of the United Kingdom (RCOphth) which recommended VKA continuation to avoid the risk of stroke and death. Importantly, 98% of surgeons adhered to the RCOphth guidelines regarding the necessity of INR measurement preoperatively. Interestingly, VKA-interrupting physicians used different interruption periods before surgeries, which were not explicitly indicated in the relevant guidelines.

Regarding glaucoma surgery, physicians’ attitudes and practices were conflicting. In a cross-sectional study in the United Kingdom, Alwitry et al^19^ received 64 completed surveys (out of 93) from a sample of glaucoma specialists. The authors found that about one-third of surgeons stopped VKA before surgeries with a mean time of 4 days (range 2-7 days). Of these surgeons, 47.6% consulted a hematologist or a general practitioner regarding VKA discontinuation, particularly for patients with MHV. Avoiding the risk of hemorrhage (more specifically, suprachoroidal hemorrhage) was the main indication of VKA stoppage. Concerning bridging therapy, heparin bridging was used by 38.1%, 14.1% using heparin bridging depending on the indication of anticoagulation, while the remainder refrained from bridging. Notably, surgeons showed significant variations in their practices regarding the timing of INR check (ranging between the day of surgery to 2 weeks before and after surgery) and the INR threshold above which surgeries were not performed.

In another study based in Brazil, Balbino et al^20^ assessed aspects of perioperative management of VKA among the members of the Brazilian Glaucoma Society (n = 52). The majority (82.7%) of respondents interrupted VKA before surgical procedures, 69.2% of them interrupted VKA 7 days before surgery, and 55.8% resumed it at the evening of the day after the procedure. However, slightly more than half of the participants (51.9%) reported AC-related hemorrhagic complications, including hyphemia, excessive subconjunctival hemorrhage, excessive postoperative bleeding, and a hemorrhagic choroidal detachment, in line with the apparent variations in physicians’ practices and the resultant complications, the authors of studies concerning perioperative AC in patients undergoing glaucoma surgeries called for an urgent need of proper guidance to control the risks of thrombosis or bleeding in these populations.

### Patients Undergoing Miscellaneous Elective Surgeries

The general clinicians’ practices regarding perioperative anticoagulation were investigated in an earlier study conducted by Oh et al.^21^ The authors sent a postal and online questionnaire to physicians who frequently involved in making relevant clinical decisions, including 4 clinical scenarios: 2 scenarios in patients with established mitral MHV who undergo either major (scenario 1) or minor (scenario 2) surgeries and other 2 scenarios in patients with established aortic MHV who undergo major (scenario 3) or minor (scenario 4) surgeries. Additionally, the survey contained different preoperative and postoperative options for VKA use and bridging. In general, the use of low molecular weight heparin (LMWH) was appreciated by most respondents in all clinical scenarios when VKA therapy must be interrupted. Although the published guidelines^22,23^ at that time have not indicated the use of perioperative anticoagulation in patients with MHV, there was no clinical consensus as revealed by the obtained responses. However, large variability in the preference of LMWH or unfractionated heparin (UFH) in high and low-risk surgeries, indicating a significant uncertainty on the optimal anticoagulation approaches and their association with the lack of proper guidance.

In another cross-sectional study,^24^ a survey was sent to physicians to assess their practices and attitudes concerning AC therapy in patients with chronic AF who would undergo elective surgeries. Clinical scenarios were classified as low or high risks of stroke. Following VKA interruption, 5 options were presented to the physicians, including bridging with full-dose UFH, outpatient full-dose LMWH, low-dose LMWH postoperatively, no bridging, or switching to another AC. Results of patients with low risk of stroke revealed that VKA interruption preoperatively and resumption after the operation was the most effective approach. In the instance of a high risk of stroke, the responses were highly variable. For example, VKA interruption, full-dose UFH, and full-dose LWMH were preferred by 54%, 24%, and 20%, respectively before the procedure. In comparison, post-procedural preferences showed an equal distribution of no bridging and in-hospital administration of full-dose UFH (35% for both). The authors stated an urgent need to unified guidelines based on robust scientific evidence, particularly for the patient at high risk of stroke.

For patients with proximal femoral fractures and long-term VKA use, the operating surgeons’ attitudes (n = 159) were assessed in a cross-sectional study conducted in the United Kingdom.^25^ In this study, the majority of respondents (75%) showed that they used either departmental or individual protocols for preoperative reversal as well as reinitiating of ACs after surgery. Additionally, 70% of them employed a “withhold and wait” approach for VKA stoppage before surgeries although this approach may be risky since delaying surgeries in patients with hip fracture might lead to deep venous thrombosis, skin breakdown, urinary tract infections and mortality.^26^ For hemiarthroplasty, most surgeons aimed for INR of <2, whereas a proportion of them considered it acceptable to proceed with the procedure with an INR up to 2.5.^25^ Of note, only 35% of the respondents in this study considered hemiarthroplasty at the agreed INR of major surgeries (>1.5). The authors suggested using low doses of vitamin K to reverse the therapeutic effects in warfarinized patients, and they underscored the lack of relevant guidelines that might assist in clinical decision making.

Flaker and his colleagues in 2016 conducted a survey for the management of periprocedural AC. They were assessing the management of AC patients undergoing surgery among different specialties, general, cardiology, gastroenterology and orthopedics surgeons. They found significant variations between respondents in identifying the high-risk patients that justify AC interruption and parenteral bridging. Additionally, they highlighted the difference between cardiologists and other specialties in performing low-risk bleeding surgeries without AC interruption. Finally, they concluded the more education for physicians in regard to the process of AC interruption are necessitated.^27^

In early 2020, a group of researchers in Qatar surveyed all HCPs from different specialties who are managing VKA peri-procedurally to assess their awareness, attitude and practice toward managing such cases.^28^ They found that practitioners’ awareness level of VKA peri-procedural management process is intermediate (64.28%). The main downward driver of these results was a low score in 3 areas. Firstly, the awareness of surgeries that do not need VKA discontinuation (response rate = 26.2%). Secondly, the awareness concerning the time at which patients must hold VKA and hold LMWH before surgery (right response rate = 42.2%, 47.1%, respectively). Thirdly, the bridging decision was an additional impediment. In bridging decision scenarios, they discovered apparent divergence in answers among departments. The study represented a broad disparity in the clinical practice of VKA periprocedural management.

## Recommendation and Conclusion

To sum up, the clinical decision regarding perioperative VKA management is a complex aspect of care. Indeed, such an issue would ultimately lead to undesirable variation in care. Individual experience and the lack of enforced structured protocols to control and assess the process make VKA periprocedural management complicated. As much as possible, there should be a unified protocol followed at the institutional level. Clinical factors should very well justify deviation from such protocols.
